# Comprehensive Survey of Clinical Trials Registration for Melanoma Immunotherapy in the ClinicalTrials.gov

**DOI:** 10.3389/fphar.2019.01539

**Published:** 2020-01-10

**Authors:** Yan-Bo Wang, Gang Lv, Feng-Hua Xu, Lin-Lu Ma, Yong-Ming Yao

**Affiliations:** ^1^ Department of Microbiology and Immunology, Trauma Research Center, Fourth Medical Center of the Chinese PLA General Hospital, Beijing, China; ^2^ Department of General Surgery, The 8^th^ Medical Centre of Chinese PLA General Hospital, Beijing, China; ^3^ Ward I of Internal Medicine, Beijing General Hospital of the Chinese People's Armed Police Force, Beijing, China; ^4^ Center for Evidence-Based and Translational Medicine, Zhongnan Hospital of Wuhan University, Wuhan, China

**Keywords:** melanoma, immunotherapy, *ClinicalTrials.gov*, trial registration, vaccines

## Abstract

**Objective:** Comprehensively evaluate the immunotherapeutic clinical trials and provide reference for melanoma treatment and research.

**Methods:** The website of *ClinicalTrials.gov* was searched to retrieve and download all registered clinical trials for melanoma immunotherapy on August 1 (updated on August 25), 2019. All registration trials met the inclusion criteria were collected regardless of the type of study, the status of recruitment, and the results of the study. The general characteristics, methodological characteristics, and the types of immunotherapeutic drugs included of these trials were analyzed.

**Results:** Finally, 242 eligible trials were included and evaluated. Of them, 30.6% were completed, 16.9% were terminated, and two were withdrawn; 77.7% recruited less than 100 participants; 30.5% were randomized; 45.5% was single group assignment; 88.8% were not masked; the primary purpose was treatment; 44.2% had data on monitoring committees; 27.7% used US FDA-regulated immunization drugs; 78.5% without results posted; 43.0% were sponsored by the industry. Immunological checkpoint inhibitors were most often studied, with 53.6% of the trials involving PD-1, the most commonly studied was Nivolumab.

**Conclusions:** Currently, most of the registered clinical trials for melanoma immunotherapy were interventional open-label trials. Most immunotherapy research hotspots were in the FDA-regulated drug product, and a few trials reported available test results. It is necessary to strengthen the supervision of results and explore and disseminate more effective and safe immunotherapy methods.

## Introduction

Melanoma is a malignant tumor originating from melanocytes and often occurs in the skin, uvea, oral cavity, intracranial, etc. (Smith et al., 2016; Tang et al., 2017; Yang et al., 2018). According to the global cancer statistics in 2018, there are more than 280,000 new cases of melanoma of skin worldwide, accounting for 1.6% of new cancer cases; and more than 60,000 cases of melanoma deaths worldwide, accounting for 0.6% of total cancer deaths (Bray et al., 2018). Although the morbidity and mortality of melanoma are not as high as other malignancies, the global burden of disease (GBD) of melanoma is increasing year by year. It is reported that the GBD of melanoma increased by 51% in 2016 compared with 2015, and the incidence of melanoma increased by 39% (Collaboration, 2018). In addition, the 5-year survival rate of advanced melanoma patients is only about 20% (Maio et al., 2015; Hamid et al., 2019). For early melanoma, it can be cured by surgical treatment; moreover, immunotherapy, radiation therapy, chemotherapy, targeted therapy, and other treatments are used for the supplementary treatment of surgery or the treatment of patients with unresectable or metastatic melanoma (Wang et al., 2018).

As a form of biological therapy, immunotherapy is widely used to treat tumors. Some researchers believe that tumor cells develop and proliferate in the tumor microenvironment, while evading the identification and clearance of the immune system in a variety of ways (Ude et al., 2018). Due to the inhibition of the production and activity of immune effector cells in the body, many signals and factors were released to the tumor microenvironment to help tumor cells spread and metastasis. Immunotherapy can stimulate immune system, activate recognition surface antigens of tumor cells by immune cells, and induce immune cells to remove tumor cells, so as to achieve the purpose of cancer treatment (Muenst et al., 2016). Up to now, many trials have been designed for immunotherapy of melanoma, including vaccines, immunomodulators, adoptive cell transfer therapy (ACT), immune checkpoint inhibitors (ICI), etc. (Koller et al., 2016). Vaccine induces immune response of immune system by active immunity, stimulates immune cells to recognize tumor specific antigens, and then destroys tumor cells. Common vaccines include dendritic cell (DC) vaccine, peptide vaccine, DNA vaccine, autologous tumor cell vaccine, etc. (Ott et al., 2014; Sarbu et al., 2017). Cytokines such as interleukin (IL)-2, IL-12, IL-15, and interferon-α (INF-α) can promote the immune recognition of melanoma and thus have function of regulating immunity (Nicholas and Lesinski, 2011). Adoptive cell immunotherapy separates lymphocytes from blood or tumor-infiltrating lymphocytes from tumors that have been surgically removed, and then transfuse them to patients after activation and proliferation *in vitro*, that to kill tumors or stimulate the anti-tumor immune effect of the body (Maus et al., 2014; Rosenberg and Restifo, 2015). In addition, immune checkpoint inhibitors (ICI), such as pembrolizumab and nivolumab, play a role in regulating the immune response of T lymphocytes in tumor microenvironment, and has made some progress in previous clinical trials (Pulluri et al., 2017).

Clinical trials of immunotherapy for melanoma continue to increase. In 1970s, the concept of “clinical trial registration” was proposed in the United States. Simes RJ (Simes, 1986) found that clinical trial with positive or promising outcomes was preferred to publish and the clinical trial registration helps to reduce this publication bias. Currently, the International Committee of Medical Journal Editors (ICMJE) requires all prospective clinical trials be registered before the first subject were included (De Angelis et al., 2004). In 2000, the *ClinicalTrials.gov* (https://clinicaltrials.gov/) was open to the public. As one of the most widely used clinical trial registration platform, its high weekly growth rates for new entries, high transparency and accessibility, and detailed information on past and present clinical trials (Ma et al., 2019), making *ClinicalTrials.gov* a representative of 16 clinical trial registry centers around the world (Zarin et al., 2017). *ClinicalTrials.gov* has received more than 300,000 clinical trials registration so far, including a number of trials on immunotherapy for melanoma. Hence, more details could be obtained from trials than those reported in final peer-reviewed publications (Cihoric et al., 2017). Moreover, harnessing the immune system for therapeutic benefit in cancer becomes an aim of immunologists and oncologists in recent years. With the development of immunotherapy for melanoma, great progress has been made, but immune-related adverse events (irAE) have also observed. Therefore, we searched and analyzed all of these trials on immunotherapy for melanoma registered in *ClinicalTrials.gov* to assess the characteristics of them and the current status of immunotherapy.

## Methods

### Data Source

We retrieved and downloaded all registered clinical trials for melanoma immunotherapy in the *ClinicalTrials.gov* website. We used its search function to search the term “melanoma” for “Condition or disease” and “Immunotherapy” for “Other terms” on August 1 (updated on August 25), 2019. Intervention (clinical trials), observation, and expanded studies were all included. Trials of open (not yet recruited, recruited) and closed (by invitation to register; active, unrecruited; suspended; terminated; completed; withdrawn; unknown) status were considered to include. There are no restrictions on the results of the study or the age of the patients enrolled. All finally included clinical trials must have a definitive record of established immunotherapy.

### Statistical Analysis

The selected records were imported into the Microsoft Excel 2007 software and all of the following information was extracted: the NCT number, status, conditions, groups or arms, experimental and control medications, sponsor, collaborators, gender, age, study phases, enrollment, funder type, study types (allocation, intervention model, masking, primary purpose, and time perspective), start date, completion date, locations, data monitoring committee (DMC), US Food and Drug Administration (FDA)-regulated product, IPD sharing statement, study documents, and study result.

The general characteristics of clinical trials were shown in descriptive statistics. The categorical data was expressed by calculating the frequency and percentage. All analyses were performed using the Microsoft Excel 2007 software.

## Results

### General Characteristics of Included Clinical Trials

**Figure 1:**
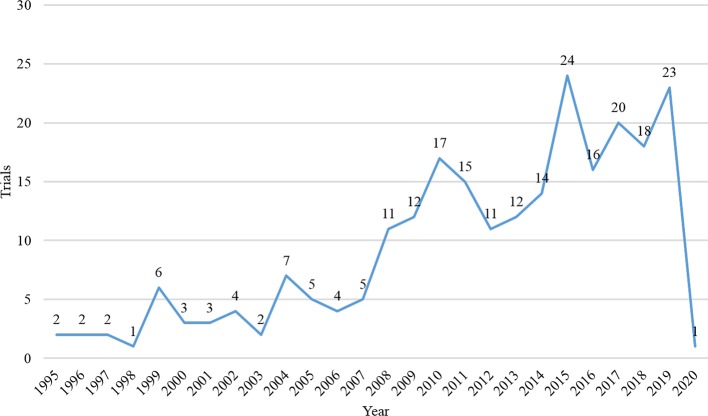
Quantity trend of registered trials per year.

A total of 395 records were identified on the *ClinicalTrials.gov*. After excluded repeated records, non-immunotherapy, and melanoma with other organ diseases trials, we finally include 242 trials. Among them, the vast majority of trials (n = 241, 99.6%) did not restrict gender of participants. Most trials (n = 224, 92.6%) were solely focused on adults, and a small number of trials (n = 18, 7.4%) were focused on both children and adults. The number of registered trials had increased significantly since 2008 (**Figure 1**), and most trials (n = 154, 63.6%) began in 2011 and beyond. The majority of trials (n = 192, 79.3%) spanned more than 24 months, and more than one-third of the trials (n = 79, 32.6%) were over 60 months. Of the eligible trials, 233 (96.3%) were interventional, eight (3.3%) were observational, and one was expanded access trial. Most of them (n = 188, 77.7%) recruited less than 100 participants, only 4.6% recruited more than 400 participants. 74 trials (30.6%) were in the completed state, followed by the recruiting state (n = 65, 26.9%); 41 trials (16.9%) were terminated (lacking funds or statistical power, business reasons, expired commitment) and two were withdrawn (no patients were enrolled). **Table 1** presented the detailed information.

**Table 1:** General characteristic of included trials (n = 242).

Name	Detail	Number	Percent
*Gender*			
	All	241	99.6%
	Not provided	1	0.4%
*Age*			
	Adult	224	92.6%
	Child and adult	18	7.4%
*Start Date*			
	Prior to 2000	16	6.6%
	2001–2010	70	28.9%
	2011–2020	154	63.6%
	Not provided	2	0.8%
*During Date*			
	0–12 months	7	2.9%
	13–24 months	27	11.2%
	25–36 months	36	14.9%
	37–48 months	38	15.7%
	49–60 months	39	16.1%
	60-	79	32.6%
	Not provided	16	6.6%
*Study Type*			
	Interventional	233	96.3%
	Observational	8	3.3%
	Expanded access	1	0.4%
*Enrollment*			
	0–100	188	77.7%
	101–400	35	14.5%
	401~	11	4.6%
	NP	8	3.3%
*Status*			
	Active, not recruiting	39	16.1%
	Completed	74	30.6%
	No longer available	1	0.4%
	Not yet recruiting	9	3.7%
	Recruiting	65	26.9%
	Suspended	3	1.2%
	Terminated	41	16.9%
	Unknown status	8	3.3%
	Withdrawn	2	0.8%

### Methodological Quality of Included Clinical Trials

Among the 233 interventional trials, 95.5% were in Phase I to III. 71 (30.5%) were randomized, while 68 (29.2%) were non-randomized. The most common intervention model was single group assignment (n = 106, 45.5%), followed by parallel assignment (n = 101, 43.4%). Most of them (n = 207, 88.8%) were not masked, only eight (3.4%) were double masked, three (1.3%) were triple masked, and four (1.7%) were quadruple masked. Most (n = 227, 97.4%) commonly adopted primary aim were treatment. In addition, eight observational trials were cohort design, including five (62.5%) prospective design and three (37.5%) retrospective design. **Table 2** presented the detailed information.

**Table 2:** Design data of included trials (n = 242).

Study Type	Study Design	Number	Percent
Interventional		233	96.3%
	*Phases*		
	·Phases 1–3	231	95.5%
	·Phase 4	1	0.4%
	·Not applicable	1	0.4%
	·Not provided	9	3.7%
	*Allocation*		
	·Randomized	71	30.5%
	·Non-randomized	68	29.2%
	·Not provided	94	40.3%
	*Intervention Model*		
	·Crossover assignment	4	1.7%
	·Factorial assignment	1	0.4%
	·Parallel assignment	101	43.4%
	·Sequential assignment	7	3.0%
	·Single group assignment	106	45.5%
	·Not provided	14	6.0%
	*Masking*		
	·None	207	88.8%
	·Double	8	3.4%
	·Triple	3	1.3%
	·Quadruple	4	1.7%
	·Not provided	11	4.7%
	*Primary Purpose*		
	·Diagnostic	1	0.4%
	·Prevention	1	0.4%
	·Treatment	227	97.4%
	·Other	3	1.3%
	·Not provided	1	0.4%
Observational			
	Cohort	8	3.3%
	·Prospective	5	62.5%
	·Retrospective	3	37.5%
Expanded Access		1	0.4%

### Detailed Characteristics of Included Clinical Trials

**Figure 2:**
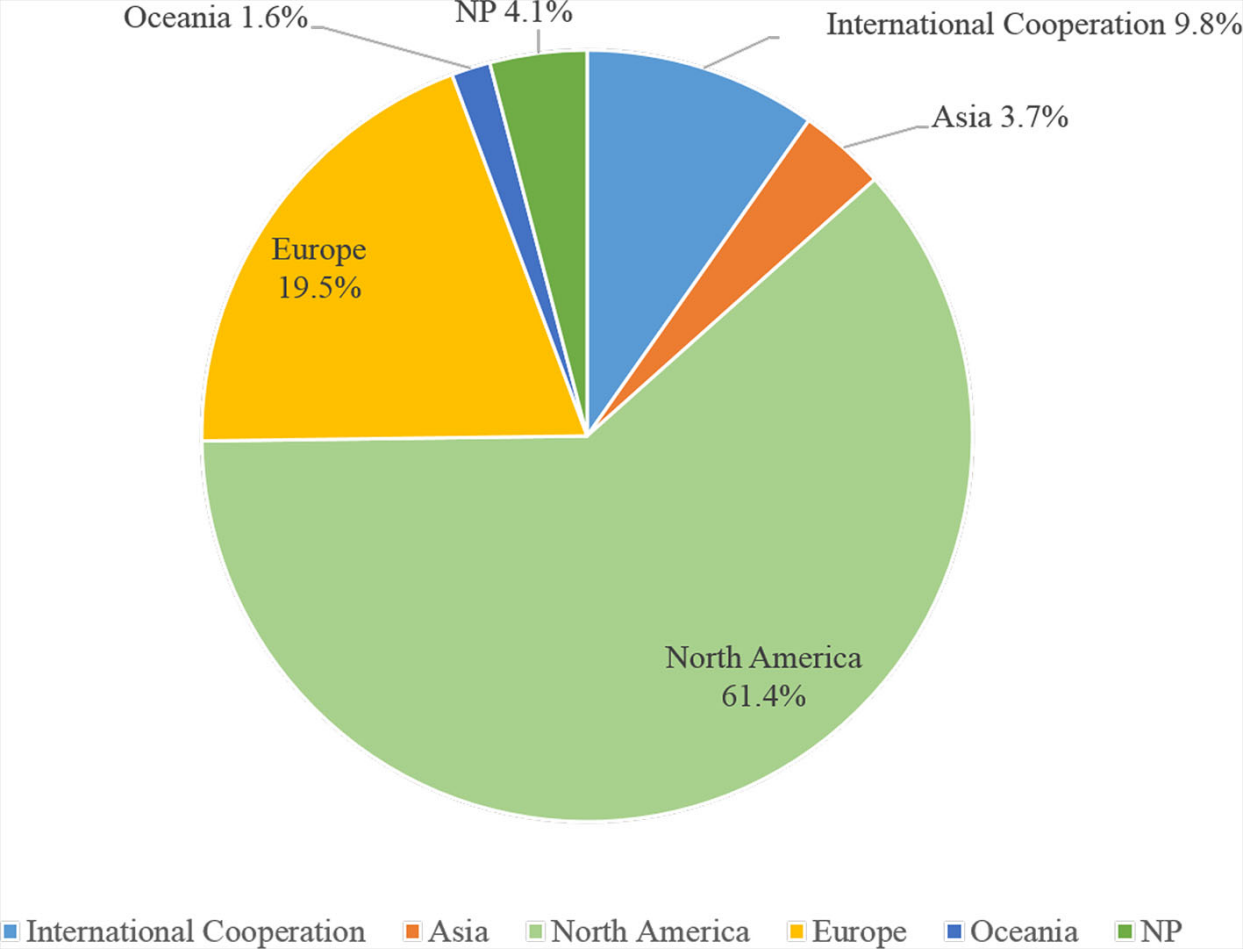
Regional pie chart for included clinical trials.

In 242 trials, less than half (n = 107, 44.2%) had DMCs, 27.7% used immunization drugs were the US FDA-regulated products, eight trials (3.3%) had IPD sharing statement, seven trials (2.9%) had results submitted, 45 (18.6%) posted results on *ClinicalTrials.gov*, and 78.5% without any results posted. Nearly half of the trials (n = 114, 47.1%) had collaborations. 104 trials (43.0%) were sponsored by the industry, less than one-third (n = 66, 27.3%) were funded by the NIH, and 16.1% were funded only by NIH (**Table 3**). 148 trials (61.2%) were conducted in North America, then in the Europe (n = 47, 19.4%), and 9.9% were based on international cooperation (**Figure 2**).

**Table 3:** Detailed characteristics of included trials (n = 242).

Name	Detail	Number	Percent
*Data Monitoring Committee*			
	Yes	107	44.2%
	No	93	38.4%
	Not provided	42	17.4%
*U.S. FDA-regulated Product*			
	Yes	67	27.7%
	No	26	10.7%
	Not provided	149	61.6%
*IPD Sharing Statement*			
	Yes	8	3.3%
	No	41	16.9%
	Undecided	19	7.9%
	Not provided	174	71.9%
*Results*			
	Results submitted	7	2.9%
	Posted on ClinicalTrials.gov	45	18.6%
	No results posted	190	78.5%
*Collaborators*			
	Yes	114	47.1%
	No	128	52.9%
*Funder type*			
	NIH	39	16.1%
	Industry	49	20.3%
	Industry and (NIH+Other)	55	22.7%
	Other	72	29.8%
	NIH and other	27	11.2%

NIH, the National Institution of Health.

### Description of Immunotherapies in Included Clinical Trials

All included trials involved four categories of immunotherapy: ACT, ICI, immunomodulators, and vaccine (**Table 4**). Among them, ICI was the most frequently (n = 155, 42.8%), followed by vaccine (n = 83, 22.9%). Among the ICI, the most studied were PD-1 (n = 83, 53.6%), followed by CTLA-4 (n = 64, 41.3%). The most studied single drug in PD-1 was nivolumab, followed by pembrolizumab. The most studied single drug in CTLA-4 was ipilimumab, and one study was tremelimumab. Among the vaccines, peptide vaccine was the most frequently studied vaccine, followed by DC vaccine, and then Autologous Tumor Cell vaccine. Among the immunomodulators, cytokines were the most widely studied, especially IL-2 (n = 50, 76.9%).

**Table 4:** Descriptions of immunotherapies in clinical trials.

Immunotherapies	Type of Drugs	Name of Drugs	Number	Percent
ICI			155	42.8%
	PD-1		83	53.6%
		Nivolumab^*^	50	
		Pembrolizumab^*^	30	
		Spartalizumab^*^	2	
		Camrelizumab	1	
	CTLA-4		64	41.3%
		Ipilimumab^*^	63	
		Tremelimumab	1	
	PD-L1	Atezolizumab^*^	5	3.2%
	LAG-3	Relatlimab	3	1.9%
Immunomodulators			66	18.2%
	Cytokine		58	
		IL-2^*^	50	
		IFN-α-2b	3	
		IFN-γ	1	
		IL-12	1	
		IL-15	1	
		rIL-21	2	
	TLRA	TLRA	1	
	Oncolytic viral	Oncolytic viral^*^	6	
	Not provided	Not provided	1	
Vaccine	Vaccine		83	22.9%
		ATC	12	
		BCG	2	
		DNA	5	
		DC^*^	21	
		Dinitrophenyl	2	
		RNA	4	
		Viral	3	
		Peptide	34	
ACT			58	16.0%

*U.S. FDA-regulated drug product; TLRA, toll-like receptor agonist; ATC, autologous tumor cell.

## Discussion

This study comprehensively analyzed drug trials registered on *ClinicalTrials.gov*, all of which explored immunotherapy and common adverse reactions to melanoma. Through analysis, we found most of the trials were interventional trials, and one third trials had been completed. Most interventional trials were phase 1–3, small sample size, and single group assignment, not blinded, for therapeutic purposes. At the same time, nearly half of the trials included the data monitoring committee, and one fifth submitted and published results. ICI and vaccine were the most widely studied immunotherapies, of which ipilimumab, nivolumab, and IL-2 were the most single drug widely studied.

Almost all subjects in these trials were gender-neutral, and more than 92% of the trials included only adults. Even though women diagnosed with cutaneous melanoma have a survival advantage due to the effects of sex hormones, there is no difference in overall survival rates between men and women (Enninga et al., 2017). Although melanoma often occurs in adults, it is also the most common skin cancer in children (Dunn et al., 2018). According to these registrations, only a few trials have been included children; obviously, there is still a great shortage of research on melanoma in children. Hence, we recommend researchers expand scope of the population in future clinical trials to get more clinical data for children with melanoma. Since 2008, more than 10 trials have been conducted each year, and 63% of trials have been carried out after 2010. The registration of clinical trials helps to increase the sharing of information of clinical trials, increase the openness of the research process, and reduce publication bias (Aslam et al., 2013). In 2004, the ICMJE issued a statement requesting that prospective clinical trials need to be registered prior to inclusion in patients (De Angelis et al., 2004). From this study, we found the number of clinical trial registrations for melanoma immunotherapy has increased significantly compared to before after 2004, this may be related to this publication policy.

More than 96.3% were interventional studies, and among them only 30.5% clearly indicated random allocation was used, 43.4% used parallel assignment models, and only 6.4% used double-masking, triple-masking, or quadruple-masking. Randomization is a very powerful method that can largely prevent confusion and reduce selection bias in treatment comparisons (Sessler and Imrey, 2015). The implementation of masking can bring many benefits to participant, care provider, investigator, and outcomes assessor (Schulz and Grimes, 2002). However, due to the different toxicity profiles of the comparators, the trials were difficult to perform blindly, so most of them were open label design. More than 64% of the research continued for three years or more, and 79 trials were conducted for more than five years. Because melanoma is invasive, patients with stage IV melanoma have an average survival of about eight months and a low five-year survival rate (Grob et al., 2017). Most clinical trials were still exploring the long-term survival of immunotherapy for melanoma (Faries et al., 2017; Schachter et al., 2017). According to result of enrollment, the sample size of most studies was still small, and 77% included fewer than 100 patients. The sample size affects population mean, variance, statistical power, and effect size (del Rio et al., 2014), which is directly related to the credibility of the results (Ruberg and Akacha, 2017). Therefore, this suggested that the minimum sample size should be estimated in advance in the design stage of clinical trials to meet the accuracy and reliability of statistics and ensure the reliability of results.

Most of the selected trials were conducted in North America, and 9.8% were conducted on more than two continents. The *ClinicalTrials.gov* is a database of privately and publicly funded clinical studies conducted around the world, which currently contains registration information for nearly 300,000 studies in more than 200 countries (Tse et al., 2018). 47.1% of trials had collaborators, 50% were conducted by NIH participate in sponsorship. Rare adverse events are unlikely to be found in small sample clinical studies, because the effect size may be too small to be evaluated. One way to increase the sample size is to conduct multi-center collaborative research to increase the external validity of the study (Yusuf et al., 1984). At the same time, the support of funds from sources such as the NIH provides a strong guarantee for the smooth development of multi-center research (Allareddy et al., 2014). 44.2% of the trials had DMCs, and DMC is critical to maintaining the scientific integrity of the trial, the accuracy and authenticity of the trial data, and the safety of the study participants (Filippatos et al., 2017). 21.5% of the trials submitted or posted their results, although the reporting rate had improved, but still need to adhere to the principle to provide accurate, complete, and timely information for all studies (Zarin et al., 2017). 27.7% of the trials reported FDA-regulated product, and federal law requires sponsors to submit summary results for applicable clinical trials, including those following the first phase of the FDA new drug approvals to *ClinicalTrials.gov* for public releasing (Schwartz et al., 2016).

The included trials were classified according to the type of immunotherapy. The results showed that most studies explored ICI and vaccines, among the ICI, the most studied were PD-1, followed by CTLA-4. The most studied single drug in PD-1 was nivolumab, followed by pembrolizumab. The most studied single drug in CTLA-4 was ipilimumab. Immunological checkpoint inhibitors (ICI) have greatly changed the treatment of advanced skin melanoma and gradually replaced traditional chemotherapy, showing great potential for the treatment of melanoma. Common ICIs include cytotoxic T-lymphocyte-associated antigen 4 (CTLA-4) antibodies (O'Day et al., 2007), programmed cell death protein 1 (PD-1) antibodies (Topalian et al., 2012), PD-L1 antibodies (Sullivan et al., 2019), and lymphocyte-activation gene-3 (LAG-3) antibodies (O'Day et al., 2007). CTLA-4 and PD-1 downregulate T cell response in lymphoid tissues and tumor microenvironments. And their monoclonal antibodies can interfere with this pathway and promote the activation of anti-cancer T cells (Levine et al., 2017). PD-L1 binds to PD-1 on T cells, which down-regulates T cell activity, and PD-L1 antibodies achieve anti-tumor effects by interfering with this pathway (Zou et al., 2016). LAG-3 is another important immunological checkpoint, and co-expression of PD-1 is associated with T cells exhaustion (Grosso et al., 2009; Park and Cheung, 2017). Common monoclonal antibodies corresponding to these four types of ICI are ipilimumab (NCT00324155), nivolumab (NCT01585194), atezolizumab (NCT03175432), and relatlimab (NCT03743766).

Vaccine is a hot spot drug and made some progress in the immunotherapy of melanoma. Among the vaccines, peptide vaccine was the most frequently studied vaccine, followed by DC vaccine, and then autologous tumor cell vaccine. Most cancer vaccines are designed to activate tumor-specific CD8^+^ cytotoxic T cells, so the most common peptide vaccination strategy is based on MHC class I-restricted peptide epitopes on TAA (Butterfield, 2015). DCs are the most effective antigen presenting cell in the immune system and have the unique ability to induce the differentiation of naive T lymphocytes into effector T cells, which have specific cytotoxic activity against a variety of antigens, including antigens expressed by tumor cells (Anguille et al., 2017). The principle of DC vaccine preparation is to collect lymphocytes from peripheral blood, induce them into DC *in vitro*, and present tumor antigens to DCs, thereby providing a large number of these cells for active immunotherapy (Dannull et al., 2013). Tumor antigen presentation to DC can be accomplished in a variety of ways (Osada et al., 2015; Wei et al., 2016). A new method was developed to present autologous tumor antigens to the cytoplasm of DCs. This method is more effective than conventional foreign aid loading. In the mouse melanoma model, the new method produces DC vaccines that show more excellent effect (Hardin et al., 2018). Geskin *et al.* conducted the efficacy of three MODC vaccines for the treatment of metastatic melanoma, differing only in the antigen loading method of autologous tumors: co-culture, fusion, or lysate pulse, and found vaccines to be safe with few side effects (Geskin et al., 2018). Other common vaccines such as recombinant vaccinia virus, plasmid DNA vaccine, autologous tumor cell vaccine, and dinitrophenyl (DNP)-modified melanoma vaccine are still in the stage of clinical trials. Although monotherapy with these vaccines is unlikely to produce substantial complete remission or cure rates in metastatic melanoma, the use of these vaccines to promote anti-tumor immunity may be an important method of future combination therapy (Wolchok et al., 2013).

Immunomodulators are an important part of melanoma immunotherapy (Nicholas and Lesinski, 2011). Common immunomodulators are cytokines such as IL-2, IL-12, IL-15, and IFN. These cytokines can help lymphocytes to recognize melanoma and achieve the purpose of treating tumors (Marabondo and Kaufman, 2017; Mirjacic Martinovic et al., 2017). IFN-α and IL-2 have been used in the immunotherapy of melanoma for decades (Buchbinder and McDermott, 2014). High-dose IL-2 is one of the first immunotherapeutic drugs to demonstrate initial clinical efficacy in advanced cancer patients (Atkins, 2006). The US-FDA approved it in 1996 for the treatment of metastatic malignant melanoma (MM). However, due to the high toxicity of HD IL-2, it is rarely used in clinical trials to treat MM patients (Ye et al., 2014). Davar *et al.* (Davar et al., 2017) retrospectively analyzed data from 237 patients receiving high-dose (HD) IL-2 from 1992 to 2015. The results showed that the overall response (OR) was 18.1% and complete response (CR) was 8.0%. The median overall survival (OS) was 64.9 months. In addition, this study found that pre-treatment level of lactate dehydrogenase (LDL) and sites of metastatic disease may be useful markers for patients who benefit from HD IL-2 therapy. The anti-tumor effect of IFN-α is expected to be induced by CD8+ T cell-mediated autologous tumor cell lysis. High-dose IFN is currently the standard adjuvant therapy, despite the high incidence of adverse events (Espinosa et al., 2016). The significant clinical efficacy of oncolytic virus (Andtbacka et al., 2015) and toll-like receptor agonist (Mauldin et al., 2015) had opened up a new path for melanoma immunotherapy, and follow-up clinical research is underway. It is expected that it will have better clinical research results and be used in clinical practice as soon as possible.

ACT is another hot spot in immunotherapy for melanoma. Common adoptive cells include tumor infiltrating lymphocytes (TIL) (Lee et al., 2016), chimeric antigen receptor modified T cells (CAR-T) (Wiesinger et al., 2019), and T cell receptor (TCR) gene modified T cells (Lagisetty and Morgan, 2012). TIL is a lymphocyte isolated from tumor tissue. After induction by interleukin-2 *in vitro*, TIL can be amplified in large quantities (Itoh et al., 1986). A clinical trial of TIL treatment for melanoma was followed up to 17 years. The results showed that the major adverse events experienced during treatment were transient and reversible, with no grade 3/4 toxicity or drug-related death observed. The recurrence-free survival of the TIL group was 14 months and nearly 4 months longer than the control group (Khammari et al., 2014). CAR-T cells are a promising approach in adoptive cell therapy for melanoma. The technique requires screening a monoclonal antibody that specifically recognizes certain tumor antigens, and then coupling the binding region of the antibody to certain peptide chains on the T cell surface membrane molecule to construct a chimeric antigen receptor; then, it is introduced into the patient's T cells for expression, and its ability to specifically recognize the antigen is activated to exert an anti-tumor effect (Firor et al., 2015; Ogba et al., 2018). One study has shown a way to stabilize the production of CAR-T cells (Wiesinger et al., 2019). In addition, genetic modification of T cells by altering the specificity of TCR is another strategy of ACT. The antigen specificity of T cells can be manipulated by genetic modification and targeted to antigens expressed by tumors. The production of tumor-specific TCR requires identification of target sequences in advance, then tumor-specific T cells are isolated from patients with tumor remission, and the reactive TCR sequences are transferred to T cells from another patient (de Witte et al., 2006). The tumor killing activity can be enhanced by altering the sequence of TCR to T cells *in vitro* to increase the strength of interaction of TCR with antigen (Robbins et al., 2008; Sharpe and Mount, 2015).

Immunotherapy has revolutionized the treatment of cancer. At the same time, given this growing success, treatment response rates, duration of treatment, why patients respond or not, and if combined with different immunotherapy will overcome this lack of response, delay acquired resistance and increase (Cooper et al., 2014). There are major limitations and unresolved issues in terms of opportunities for success. Given the complexity of immune activation and the considerable variability of tumor biology in patients and tumor types, it is necessary to understand the body's immune pathways, the molecular and immune basis of the disease, and develop interventions and combinatorial strategies that are more suitable for the treatment of cancer patients. Explore patient choices and biomarkers (Ingles Garces et al., 2019). Although immunotherapy has shown promising success, further and ongoing research is needed to determine safety, efficacy, optimal combination, dosage, and timing. Our study also has some limitations. This study only retrieves trials in the *ClinicalTrials.gov*, although approximately two-thirds of total global registrations, we might miss some trials registered in other 15 registration centers (Zarin et al., 2017) that were not fully evaluated. All information is obtained from the *ClinicalTrials.gov*, and some information of registration trials that has not been submitted to the website, therefore, some studies cannot be fully evaluated.

In conclusion, up to now, most clinical trials related to melanoma immunotherapy registered in the *ClinicalTrials.gov* were interventional trials; and although the number of registered studies increases gradually every year, the number of registered trials was still small. At the same time, it is encouraged to register on the clinical trial registration platform. In addition, we noticed that the results of some clinical trials were not uploaded to registration platform after the end of the trial. It is suggested that the researchers of clinical trials update the latest results of the trial regularly, which will help disseminate information in this field and help doctors get the research frontier as soon as possible. Although some adverse reactions may occur in the course of immunotherapy for melanoma, as an effective treatment for melanoma and even other malignant tumors, we should increase our energy and financial investment in the exploration of immunotherapy.

## Data Availability Statement

The raw data supporting the conclusions of this article will be made available by the authors, without undue reservation, to any qualified researcher.

## Author Contributions

Y-MY designed this study. Y-BW and GL performed search and collected data. F-HX re-checked data. L-LM and GL performed analysis. Y-BW wrote the manuscript, Y-MY reviewed the manuscript.

## Conflict of Interest

The authors declare that the research was conducted in the absence of any commercial or financial relationships that could be construed as a potential conflict of interest.
